# Effect of chamomile aromatherapy with and without oxygen on pain of women in post cesarean section with spinal anesthesia: A randomized clinical trial

**DOI:** 10.1016/j.heliyon.2023.e15323

**Published:** 2023-04-11

**Authors:** Hajar Zamani Habibabad, Ardashir Afrasiabifar, Afshin Mansourian, Mahboubeh Mansourian, Nazafarin Hosseini

**Affiliations:** a Student Research Committee, Yasuj University of Medical Sciences, Yasuj, Iran; b Professor of Nursing, Yasuj University of Medical Sciences, Yasuj, Iran; c Assistant Professor of Department of Anesthesiology, Yasuj University of Medical Sciences, Yasuj, Iran; d Medicinal Plants Research Center, Yasuj University of Medical Sciences, Yasuj, Iran; e Social Determinants of Health Research Center, Yasuj University of Medical Sciences, Yasuj, Iran

**Keywords:** Aroma therapy, Oxygen, Cesarean section, Spinal anesthesia, Pain, Chamomile

## Abstract

**Background:**

Pain is the most common side effect in cesarean section with spinal anesthesia. It seems that oxygen therapy and chamomile aromatherapy may diminish pain. The present study was conducted to examine and compare the effect of chamomile aromatherapy with and without oxygen on the severity of pain of women following cesarean section surgery with spinal anesthesia.

**Methods:**

The present randomized clinical trial study was carried out on 136 women undergoing cesarean section surgery with spinal anesthesia at Imam Sajad Hospital, Yasuj, Iran in 2020. The eligible women were assigned into four 34-member groups including oxygen therapy plus aromatherapy, oxygen therapy, aromatherapy, and control via block randomization. Each of these interventions were performed 6, 6.30 and 7 h post operation. In the first intervention group, one drop of chamomile essential oil with distilled water was poured into a small nebulizer using a simple mask connected to 6 L of oxygen. The second intervention group received oxygen without chamomile aromatherapy at similar times, and the third intervention group received chamomile aromatherapy without oxygen. The control group received only routine interventions. The instrument used in the research was visual analog scale which was completed by the researcher 6, 12, 18 h after cesarean section. The data were analyzed by the SPSS software version 20.

**Results:**

There was a significant difference in the intensity of pain of patients between the various groups of study following the interventions (P < 0.001). Pain intensity reduced significantly in the group receiving combined intervention of chamomile aromatherapy plus oxygen compared to the other three groups. Moreover, the pain intensity diminished more in the groups undergoing each of these interventions alone as compared to the control group (P < 0.05).

**Conclusions:**

The combined intervention of chamomile aromatherapy with oxygen was more effective than each of the chamomile aromatherapy and oxygen interventions alone in reducing the pain of cesarean section patients though each intervention alone was still effective in lowering pain post-operation.

## Abbreviation

VAS: Visual analog scale
WHO: World Health Organization
FDA: Food and drug administration
NSAIDs: Non-steroidal anti-inflammatory drugs
COX-2: Cyclooxygenase-2
C-section: Cesarean-section

## 1 Introduction

The World Health Organization (WHO) has recommended 10–15% Cesarean section (C-section) rate for live births [1]. Nevertheless, this value has been reported 48% for Iran [2]. C-section with spinal anesthesia is associated with side effects including pain, hypotension, nausea and vomiting, as well as respiratory distress [3]. Furthermore, due to impairment in the normal perfusion, surgical wounds have oxygen deficiency, where local ischemia creates wound, acidosis, and pain at site of incision [4]. In a study by Borges et al. the intensity of pain in C-section was 92.7% [5]. For pain treatment, non-steroidal anti-inflammatory drugs (NSAIDs) and narcotics are used [6], which may ensue side effects such as drowsiness, addiction, respiratory distress, and toxic metabolic [7]. Faster pain alleviation could contribute to faster breastfeeding to the newborn, early bed discharge, prevention from atelectasis, prevention from deep vein thrombosis, and early intestinal functioning [8].

WHO recognizes the significant role of alternative and complementary medicine in the treatment and prevention from chronic diseases and improving the quality of life. One of these supplementary treatments is aromatherapy. Aromatherapy refers to the application of aroma extracted from plants in the enhancement of physical, psychological, spiritual health as well as maintaining health [9]. It is used locally, inhalatory, with bathing, and through massaging [10,11]. The scent resulting from aromas changes into a nerve impulse which migrates to the olfactory bulb and then reaches the limbic region of the brain, causing release of neurotransmitters including encephalin, endorphin, noradrenaline, and serotonin [9,12,13]. This leads to a sense of relaxation, stress reduction, pain alleviation, and eventually psychological and physical changes [14]. Among aromatic herbs, chamomile with the scientific term of Matricaria chamomila has been registered in the herbs of the world and recommended for pharmacotherapies [15]. The Food and Drug Administration (FDA) classifies the chamomile essential oil as a safe drug [16,17]. The chamomile essential oil has compounds including chamazulene as a sesquiterpene aromatic chemical compound belonging to the terpenes with anti-inflammatory and antioxidant properties, and bizabolol alpha as a natural monocyclic sesquiterpene alcohol with anti-inflammatory and digestive effects [18]. Saghafi (2018) indicated that essential oil of chamomile was safe and effective for controlling the pain of women suffering from mastalgia [19].

In a study by Najafi et al. chamomile Flower Essence inhalation caused a reduction in postoperative pain in women undergoing elective caesarean section under spinal anesthesia [20]. Moreover, Aradmehr et al. in the study, revealed that chamomile cream effectively reduced episiotomy pain in women [21]. Moreover, a study by Modarres et al. indicated that chamomile capsules reduced menstrual pain intensity [22]. On the other hand, the results of the study exposed that the process of episiotomy repair was the same between the chamomile and the control groups [23].

In several animal models, oxygen therapy reduced pain [4]. Oxygen supplements similarly double the oxygen in the tissue. Combining oxygen and better perfusion may reduce pain by reducing local acidosis. Oxygen is both cheap and safe and could be combined with other treatments and applied several times a day [24,25]. In a systematic review, more than 75% of headaches alleviated with normobaric oxygen therapy, 117 of 150 attacks eased with 75% oxygen, and 30 of 148 attacks improved with 20% oxygen [26]. A study by Yagishita (2017) indicated that hyperbaric oxygen could reduce inflammation and ankle pain in athletes [27]. Nonetheless, in the study by Cohen et al. Hyperoxia did not significantly improve postoperative pain [4].

The results of the mentioned studies indicated a contradiction in the effect of oxygen and chamomile aromatherapy on pain. Furthermore, there is the lack of study on the combined effect of the two on pain. Therefore, the present study was conducted to determine and compare the effect of chamomile aromatherapy with and without oxygen on the pain of women post C-section with spinal anesthesia.

## 2 Materials and methods

### 2.1 Study design

The present randomized clinical trial study was a parallel-controlled trial study registered on Iranian Registry of Clinical Trials under the code IRCT20141222020401N7. The study was conducted on 136 women referring to Imam Sajjad hospital of Yasuj, Iran, for elective C-section with spinal anesthesia from January 05, 2020 to march 18, 2020. The research population consisted of women hospitalized in the obstetrics ward of the hospital due to non-emergency cesarean section with spinal anesthesia.

In the present study, interventions included chamomile aromatherapy, oxygen, and the combination of chamomile essential oil with oxygen, and the pain was the result. Control and tree intervention groups participated in this study, and the pain at 6 (baseline), 12, and 18 h’ post-operation were compared between and within the study groups. The intervention was single-blind. Only statistical analyzers did not know in which group they were.

### 2.2 Sampling and participants

The Participants consisted of women referring to Imam Sajjad hospital of Yasuj, Iran for elective C-section with spinal anesthesia who were eligible and had inclusion criteria.

The sample size was determined as 34 in each group and 136 individuals overall based on the parameters of α = 0.5, 1-α = 0.95, $Z_{1-\frac{\alpha }{2}=1.96}\text{,}$ β = /2, 1-β = /8, $Z_{1-\beta =/85}$ using the following formula:

$$n=\frac{\left[\left(z_{1-\frac{\alpha }{2}}+z_{b}\right){\;}^{2}*\left(\bar{p}\right)\left(1-\bar{p}\right)\right]}{\left(p_{1}-p_{2}\right){\;}^{2}}$$

A total of 136 participants were chosen by the researcher through available sampling and then assigned into four groups intervention 1 (chamomile aromatherapy with oxygen), intervention 2 (oxygen), intervention 3 (chamomile aromatherapy), and control group (n = 34 in each group) through block randomization method by the researcher, as follow: Labels A, B, C, D were written on four sheets, and the names of each of the research interventions were also written on four separate sheets, and a colleague who did not know about the study was asked to put a label for each intervention randomly. Hence, due to the existence of four groups in the study, twenty-four blocks were obtained based on the four-factorial law, the size of each block was different, and the order of their placement was different. At that point, the blocks were randomly selected by placement, and after determining the order of placement of the patients in each block, the patients were assigned to each group. The selection of blocks continued until 34 samples were placed in each group.

The inclusion criteria were as follows: volunteers of elective C-section with spinal anesthesia who had no problem regarding spinal anesthesia according to the physician, and volunteered to participate in the study. Exclusion criteria included sensitivity to chamomile, any middle-ear disease, pregnancy toxicity, asthma, acute or chronic diseases, allergy and olfactory problems, smoking cigarette and consuming alcohol.

## 3 Data collection instrument

The data were collected using a form which included data such as age, body mass index, number of pregnancies, duration of surgery, pre-operation hemoglobin, and Spo2 in the ward [28]. The data collection form was completed by the researcher by interviewing the participants and using the information in the patients' files. Pain intensity (0–10) [29] was measured using Visual Analog Scale (VAS) which is in the form of a numerical ruler. Spo2 was measured using pulse oximetry device.

In different Iranian and international studies, the reliability and validity of the VAS has been confirmed. VAS is a valid and standard instrument applied in different countries on various groups with different types of patients [[29], [30], [31], [32]].

### 3.1 Intervention

Necessary instructions were given to three nurses who cooperated with the researcher in conducting research interventions. The day prior to the operation, the list of C-section operations of the next day was prepared and the inclusion criteria for patients present in the list were examined through asking questions from patients. The measurement outcome was pain intensity of the site of C-section. Each group of the patients were studied in separate groups depending on the group they were in (control or intervention); four rooms were considered for this purpose. After entering the operation room, the patient was located on the operating room bed and the monitoring device was connected to the patient. The patients were prescribed 400 cc of intravenous normal saline. At that point the patient was put in a sitting position and after preparation and drep of the anesthesia site, spinal anesthesia was performed at the L4-L5 intervertebral level with the Quincke spinal needle model, No. 25 of Dr-J company. 12.5 mg of 5% bupivacaine (2.5 cc) of Cobel Darou company was injected intrathecally. Then the patient was positioned in a supine position and the level of anesthesia was brought to the T8 vertebral level with the help of the tilt of the operating room bed. Afterwards, the surgeon was permitted to perform the operation. No analgesics were used during surgery. Nonetheless, in the recovery room, after the operation, 500 mg of Apotel of Cobel Darou was injected to all patients.

Each intervention was completed three times: 6, 6.30, and 7 h post operation, and each time for 5 min by the first author (nursing student in MSc).

#### 3.1.1 Intervention group 1

Chamomile aromatherapy with oxygen: Chamomile essential oil was purchased from Zardband Company in Iran. At the time of entrance to the obstetric ward post-operation, one drop of chamomile essential oil was rubbed on the wrist of each patient participating in this group in order to diagnose any possible sensitivity (patch test) so that in case of sensitivity the patients would be excluded. In this group, one drop of chamomile essential oil plus 3 cc distilled water was poured into a small nebulizer connected to a simple mask [33], with the oxygen being adjusted at 6 L/min [34].

#### 3.1.2 Intervention group 2

The participants of this group received 6 L of oxygen per minute with simple mask without chamomile aromatherapy.

#### 3.1.3 Intervention group 3

Following the patch test, they received chamomile aromatherapy with nebulizer and without oxygen.

#### 3.1.4 The control group

Received only routine interventions. Note that all the four groups received therapeutic interventions and the common recommended interventions in the ward.

All C-section patients received diclofenac sodium 100 mg suppository according to the ward's routine immediately after entering the ward. Moreover, Narcotics or Diclofenac suppository and preferably Diclofenac suppository was prescribed as PRN (Pro Re Nata) by the physician for each patient in all the study groups, which was given if only requested by the patient.

During anesthesia, recovery, and in the ward, the blood oxygen saturation level was measured using finger pulse oximeter, and then recorded in the data collection form. The intensity of pain was measured and recorded in the data collection form using VAS at times of 6 (baseline), 12 and 18 h post-operation.

### 3.2 Statistical analysis

The normality of data in the groups was initially evaluated using the Kolmogorov-Smirnov test. Regarding the normal distribution of the data, the parametric Analysis of Variance (ANOVA) and non-parametric Median and Friedman tests were used as well. Moreover, post hoc multiple comparison test with Bonferroni correction to follow the differences between the groups was used. The p-value in this study was considered less than 0.05.

### 3.3 Ethical considerations

The ethics code with the number of IR.YUMS.REC.1398.036 on 2019-06-25 was granted by the ethics committee of research at Yasuj University of Medical Sciences. Written informed consent was obtained from the patients and their spouse. The research interventions, process of spinal anesthesia, side effects of anesthetics and analgesics on mother and fetus were explained to all patients in the studied groups. Based on previous clinical trials, usage of oxygen and chamomile aroma with the prescribed values is safe for C-section women. The patients in the present research were not deprived of routine treatments, and none of the groups had restrictions to receive analgesics after the surgery. All methods were performed in accordance with the relevant guidelines and regulations in accordance with the Declaration of Helsinki.

## 4 Results

### 4.1 Demographic and clinical characteristic

**Fig. 1:**
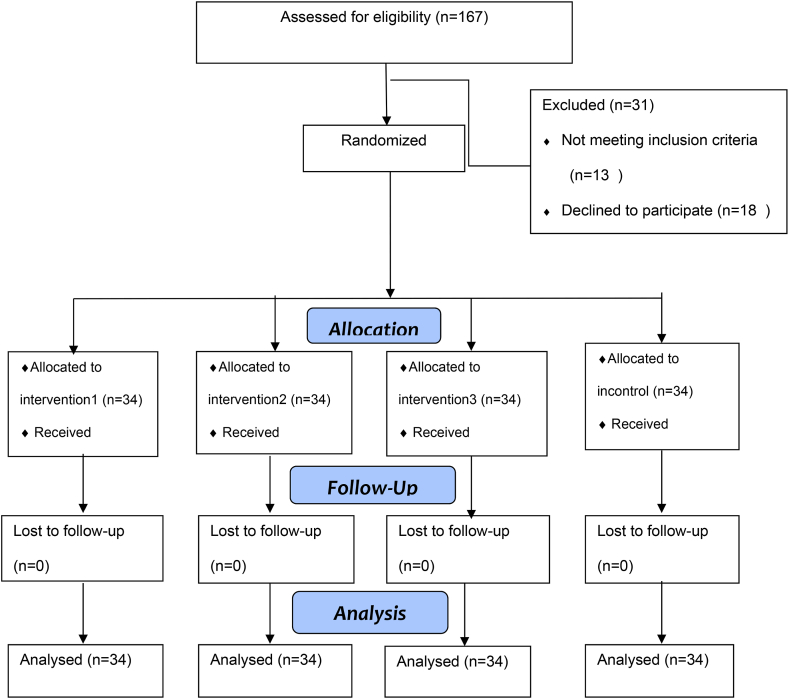
CONSORT flow diagram of participants.

The data was collected from 136 women who underwent cesarean section with spinal anesthesia. All participants remained in the study until the end of the intervention (Fig. 1). The overall mean age and duration of their surgery were 30.8 ± 5.6 (Range; 17–44 years old) and 32.3 ± 5 (Range; 25–45 min), respectively. In comparison between groups at the beginning of the study, no significant difference was reported between the four groups in mean body mass index, duration of surgery, serum hemoglobin level and Spo2 (Table 1).

**Table 1:** Demographic and clinical information of the participants in the four groups.

Groups		Age	Body mass index	Duration of surgery	Hemoglobin	Spo_2_ in the ward
Aromatherapy with oxygen		32.8 ± 5.9	26.0 ± 4.1	30.2 ± 5.0	12.8 ± 1.6	95.2 ± 1.1
Oxygen therapy		31.2 ± 3.9	26.5 ± 3.4	32.6 ± 5.9	12.7 ± 1.0	95.3 ± 1.3
Aromatherapy		29.2 ± 5.5	27.4 ± 4.3	32.3 ± 4.4	12.4 ± 1.7	95.4 ± 1.3
Control		29.3 ± 6.0	25.7 ± 3.7	33.5 ± 4.1	12.3 ± 1.3	95.7 ± 1.5
ANOVA	F	3.5	1.3	2.5	0.9	0.7
	P	0.01	0.27	0.56	0.40	0.50

Data was reported by Mean ± SD. ANOVA: Analysis of Variance.

## 5 Pain characteristics

**Fig. 2:**
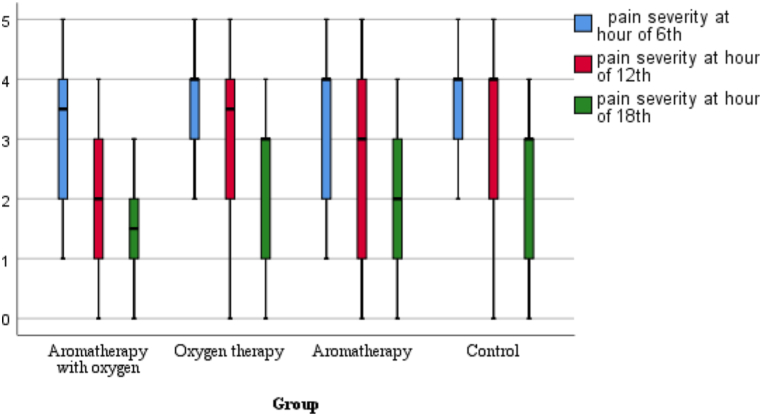
Box plot for the severity of pain by group/times.

It was indicated that the severity of pain (Fig. 2) of women after C-section surgery following all the three interventions of aromatherapy with oxygen, oxygen therapy, and aromatherapy lessened.

Between-group comparisons indicated that the four groups significantly differed from each other in median of the severity of pain at 12th and 18th hours (Table 2).

**Table 2:** Between and within group comparisons for outcome variables.

Group Outcome		Aromatherapy with Oxygen N = 34		Oxygen therapy N = 34		Aromatherapy N = 34		Control N = 34		Between-Subjects^a^
		Median	Range	Median	Range	Median	Range	Median	Range	
Pain severity	6th hour^b^	8	6–10	9	7–10	8.5	7–10	9	7–10	0.06
	12th hour^c^	6	4–8	7.5	6–9	7	5–9	8.5	7–10	0.001
	18th hour^d^	4.5	3–6	6	4–7	5.5	4–7	7.5	6–9	0.001
	Within-Subject^e^	0.001		0.001		0.001		0.001		

a Between-subject comparison using Median test.

b 6th hour post-operation.

c 12th hour post-operation.

d 18th hour post-operation.

e Within-subject comparison using Friedman's test.

Pairwise comparisons for pain variable indicated that the group of aromatherapy with oxygen significantly (p = 0.001) differed from the other three groups. Moreover, the results of pairwise comparisons correspondingly indicated that women who received aromatherapy (the aromatherapy group) or oxygen (the oxygen therapy group) significantly (p = 0.001) scored lower in the severity of pain than women in the control group; however, the two groups did not differ significantly (Table 3).

**Table 3:** Pairwise comparisons (post HOC) by median of outcome variables.

Pairwise Comparison (Group _I_- Group _J_)	Pain severity	
	12th hour^a^	18th hour^b^
Aromatherapy with Oxygen–Oxygen therapy	0.001	0.001
Aromatherapy with Oxygen- Aromatherapy	0.001	0.007
Aromatherapy with Oxygen- Control	0.001	0.001
Oxygen therapy- Aromatherapy	1	1
Oxygen therapy- Control	0.07	0.001
Aromatherapy- Control	0.003	0.001

a 12^h^ hour post-operation.

b 18th hour post-operation.

The results of the present study indicated that there were significant differences among the three study times in the severity pain scores for the all groups. Within-subject comparison using Friedman's test indicated that women on all groups significantly (p = 0.001) reported lower severity of pain at 18 h than 12 h as well as at 12 and 18 h than 6 h post-operation (Table 4).

**Table 4:** Within-subjects comparison for outcome variables.

Group Pairwise Comparison (Time _I_ –Time _J_)		Aromatherapy with Oxygen		Oxygen therapy		Aromatherapy		Control	
		Test statistic	Adj.Sig^a^	Test statistic	Adj.Sig	Test statistic	Adj.Sig	Test statistic	Adj.Sig
Pain severity	18th hour^b^ - 6th hour^c^	1.8	0.002	1.8	0.001	1.7	0.001	1.1	0.001
	18^h^ hour - 12th hour^d^	0.83	0.001	1.1	0.001	0.86	0.001	0.85	0.001
	12th hour – 6th hour	1.01	0.001	0.66	0.02	0.86	0.001	0.27	0.7

a Significance values have been adjusted by the Bonferroni correction for multiple tests.

b 18th hour post-operation.

c 6th hour post-operation.

d 12th hour post-operation.

## 6 Discussion

The effect of chamomile aromatherapy and oxygen was determined on the severity of pain among women post C-section operation with spinal anesthesia. No study was found to have explored the combined effect of chamomile and oxygen on the intensity of pain of C-section with, which highlights the novelty of the present research. At 12 h post-operation, this combined intervention reduced the pain intensity more considerably than all intervention groups and the control, though the aromatherapy intervention was also more effective on reducing pain intensity. At 18 h post-operation, all intervention groups were more effective than the control for mitigating pain intensity.

The present study displayed that aromatherapy with chamomile essential oil alone reduced the pain after the cesarean section of women. Yet, the combination of chamomile aromatherapy with oxygen alleviated the pain after cesarean section more than chamomile aromatherapy alone. On the other hand, chamomile aromatherapy did not significantly affect the pain of these patients compared to oxygen. Similar to the present results, Stea et al. reported the effect of inhaling essential oils on postoperative pain [35]. Also, the study by Najafi showed that chamomile essential oil reduced the pain of the cesarean section in women after caesarean section, and the need for analgesics was reduced [33].

Likewise, in the study by Saghafi et al. on patients suffering from mastalgia, the effectiveness of essential oil of chamomile was demonstrated in controlling mastalgia [19]. The above study and the present one displayed the effect of chamomile on pain, though the type and site of pain of the participants were different between the two studies.

Similar to the results of the present study, in Aradmehr et al.'s study, chamomile cream effectively reduced episiotomy pain in women [21]. In the mentioned study, chamomile was used for a longer duration and in the form of a cream, while in the present study, it was used on the first day of childbirth and in the form of aromatherapy. In addition, in the mentioned study, the McGill pain questionnaire was used to measure pain, but the present study used the visual analog method. Moreover, similar to the present study's results, Modarres et al. revealed that chamomile capsules reduced the intensity of menstrual pain [22]. That study, similar to the present study, indicated the positive effect of chamomile on pain, but chamomile capsules had been used*.* In addition, a study by Zargaran et al. indicated that the flavonoids of volatile chamomile oils could relieve migraine pain [36], which is consistent with the results of the present study.

The analgesic pains of chamomile may be attributed to the following reasons: (1) the effect of Chamazulene and apigenin on activated macrophages leads to the inhibition of propagation and synthesis of nitric oxide; (2) chamomile flavonoids with potent inhibitory effects against prostaglandin E2 in macrophages can play a selective COX-2 inhibitor; (3) chamomile polyphenols can reduce pain because of inhibiting anti-inflammatory biomarkers in THP_1_ macrophages which have anti-inflammatory effects [36]. It has also been demonstrated that COX-2 inhibitors can block and halt central neurons and sensitivity of peripheral meningeal receptors [37]. Thus, it can play the role of NSAIDs such as naproxen and ibuprofen without negative effects on gastrointestinal [36]. In the present study, chamomile may have applied its analgesic effects through several opioidergic, serotonergic, and inflammatory mechanisms. Various studies have proposed the analgesic potential of chamomile through opioidergic mechanism [[38], [39], [40]]. The anti-inflammatory effect of chamomile is due to inhibitory effects of chamomile essential oil especially bisabolol and chamazulene on lipoxygenase and cyclooxygenase enzymes [38].

Unlike the results of the present study, Pazandeh et al. found that the chamomile essential oil with steam distillation method did not reduce episiotomy pain [39]. In the above study, three drops were poured into plenty of water, but in the present study only one drop was poured into 1.5 cc distilled water, whereby aromatherapy was performed using a nebulizer. This difference might have resulted from differing quality and quantity of chamomile essential oil as well as differences in the type, site of wound, as well as method and time of the consumption of chamomile in the two studies.

Although 12 h of oxygen therapy did not significantly reduce the pain, but on the other hand 18 h of oxygen therapy reduced the pain of the patients after the operation. The study by Shuh-Hofer et al. indicated that oxygen by preventing plasma protein leakage in the dura mater reduced inflammation of neural tissues; thus, oxygen was effective in treating headache [20]. Furthermore, similar to the results of the present study, a study by White (2014) stated that oxygen could significantly reduce the pain associated with tourniquet in patients undergoing hand surgery under local anesthesia [40]. Although in the study mentioned above the site and type of pain were different from the site and type of C-section pain, in both studies oxygen lessened the pain. Similar to the results of the present study, Bennett et al. stated that the effect of hyperbaric oxygen on the treatment and prevention of migraine and normobaric oxygen on cluster headaches [26]. However, in the prospective study by Cohen et al. hyperoxia did not reduce postoperative pain and the need for opioids [4]. Perhaps the reason for this difference is in the type of study, a large number of samples, and the difference in the type of anesthesia in the above study (general anesthesia). In this study, 100% hyperbaric oxygen was used, but in our study, it has a small percentage of oxygen.

In the present study, oxygen and chamomile aromatherapy indicated the greatest pain mitigation followed by aromatherapy and oxygen. These results suggested that oxygen with chamomile can have synergistic effects on reducing the severity of pain at the site of C-section, though they can also alleviate pain. A study in Egypt indicated that lavender aromatherapy through oxygen mask could reduce C-section pain [41]. In the present study, aromatherapy with chamomile has been used through oxygen mask, and similar to the mentioned study above, it was effective on C-section pain mitigation. However, Davami et al.'s study indicated that chamomile and marigold ointment was effective in relieving episiotomy pain 4 h after delivery and reducing the need for sedatives [42]. In the above study, the combination of chamomile and marigold was used as an ointment. Still, the combination of chamomile aromatherapy and oxygen was used in the present study.

One of the strengths of the present study was that patients showed a greater desire to use traditional therapy such as chamomile aromatherapy or oxygen therapy for pain relief compared to analgesics, and this led to more patient cooperation in the research.

However, the present study had some limitations. Since VAS is subjective in measuring pain, the scores given by patients may have had random errors. In addition, it was necessary for the patients with pain intensity above 3 to receive analgesics (PRN). However, despite the explanation about the lack of side effects of analgesics on the newborn, the patients were not eager to receive analgesics, particularly narcotics, since they were worried about transferring them to the newborn through their milk. It would have been better to use a placebo for the patients in the control group, but it was not possible because of the smell of chamomile. Moreover, it would have been more desirable to continue the pain measurement in 24 h after the cesarian section, but it was not considered in the design of the study.

If these results are replicated in subsequent studies, the combined intervention of oxygen and aromatherapy is suggested as a cheap and side-effect free complimentary treatment for reducing pain of C-section patients undergoing spinal anesthesia post-operation.

## 7 Conclusions

The results of the present study suggest that the combined intervention of chamomile aromatherapy and oxygen had a more marked effect compared to oxygen and chamomile aromatherapy alone on reducing the pain post cesarean operation with spinal anesthesia, though each of them alone were also effective.

Further studies are needed for the effect of these interventions on pain in a larger sample size by starting interventions faster after surgery, increasing the duration and frequency of interventions, and measuring pain 24 h after cesarean section in addition to the times of the present study. Moreover, the authors suggest that in future studies, measuring other variables such as anxiety associated with pain, analgesics required by patients, nausea and vomiting, sleep quality, and patients' overall satisfaction with the treatment received following chamomile aromatherapy with and without oxygen should be assessed.

## Declaration of competing interest

The authors stated no conflict of interest.

## Funding statement

A part of this project was financially supported (Grant No. 960588) by the Vice Chancellery of Research of Yasuj University of Medical Sciences, Iran.

## Author contribution statement

Nazafarin Hossein: Conceived and designed the experiments; Analyzed and interpreted the data; Wrote the paper. Afshin Mansourian: Analyzed and interpreted the data. Mahboubeh Mansourian: Contributed reagents, materials, analysis tools or data. Hajar Zamani: Performed the experiments; Analyzed and interpreted the data; Wrote the paper. Ardashir Afrasiabifar: Conceived and designed the experiments.

## Data availability statement

Data will be made available on request.

## Declaration of interest's statement

The authors stated no conflict of interest.
